# Genetic variants in the *CD59* gene: An exploratory study of large genome databases

**DOI:** 10.1111/trf.18331

**Published:** 2025-07-30

**Authors:** Kshitij Srivastava, Thomas Christopher Recupero, Willy Albert Flegel

**Affiliations:** ^1^ Department of Transfusion Medicine NIH Clinical Center, National Institutes of Health Bethesda Maryland USA

**Keywords:** computational model, real‐world data, hemolysis, immunohematology, population genetics

## Abstract

**Background:**

CD59 is a GPI‐anchored glycoprotein on the surface of many cell types. It inhibits the assembly of the membrane attack complex, thus preventing complement‐mediated cell lysis. Lack of a functional CD59 protein causes recurrent ischemic strokes, neuropathy, and chronic hemolysis. We aimed at a comprehensive analysis of the *CD59* gene from publicly available databases to identify variants and evaluate their pathophysiologic potential.

**Methods:**

Variants in the *CD59* coding sequence of exons 4, 5, and 6 and their splice sites were systematically compiled from 4 major populations across 6 whole‐genome and whole‐exome databases. The PredictSNP algorithm assessed the functional impact of non‐synonymous variants.

**Results:**

Among 488,592 individuals, 160 distinct alleles were identified in 6881 subjects (0.7%). Among 93 alleles with non‐synonymous variants, 43 were classified as deleterious and 49 (2 variants encoded the same amino acid change) as neutral by PredictSNP. Additional variants included: 53 synonymous, 9 deletions/duplications, 3 splice site, and 2 nonsense. Among the 14 non‐synonymous variants reported in patient samples, 9 were classified as deleterious (64.3%) and 5 as neutral (35.7%).

**Discussion:**

We collated a list of *CD59* variants, which were classified as neutral or deleterious by PredictSNP, from genome databases. These results can be applied to identify individuals with possible latent CD59 deficiency symptoms, such as hemolytic transfusion reactions. In conjunction with clinical data, these *CD59* variants can guide personalized clinical decisions.

**Abbreviations**
1000GP: 1000 Genomes Project
3MAG: Three Million African Genomes
ACHE: acetylcholinesterase
AoU: all of us
CD59: cluster of differentiation 59
ClinVar: clinical variation database
ER: endoplasmic reticulum
GA100K: GenomeAsia 100K Project
GPI: glycosylphosphatidylinositol
HGDP: human genome diversity project
HLA: human leukocyte antigen
IndiGen: IndiGenomes Database
kDa: kilodalton
LoF: loss of function
MAC: membrane attack complex
MIM: mendelian inheritance in man
NCBI: National Center for Biotechnology Information
NHLBI: National Heart, Lung, and Blood Institute
NHS: National Health Service
pLI: probability of being Loss‐of‐function intolerant
PredictSNP: prediction server for single nucleotide polymorphism effects
RefSeq: reference sequence
SAGE: South Asian Genomes and Exomes
SNV/SNVs: single nucleotide variant(s)
UK: United Kingdom

## 1 INTRODUCTION

CD59 is a small glycoprotein of 20‐kDa molecular mass attached to the outside of cell membranes.^1^ It is highly expressed on the membrane of many cell types, though expression levels differ greatly between cell types.^2^, ^3^, ^4^ It protects cells from complement‐mediated cell lysis by inhibiting the assembly of the membrane attack complex (MAC) and blocking the complement terminal pathway.^5^, ^6^ A lack of functional CD59 protein has been linked to chronic hemolysis or immune‐related neuropathies^7^, ^8^, ^9^, ^10^, ^11^ and can be caused by homozygous or compound heterozygous^12^ non‐synonymous,^13^, ^14^, ^15^, ^16^, ^17^, ^18^ frameshift,^15^, ^19^, ^20^, ^21^, ^22^, ^23^, ^24^ splice site^24^ and nonsense variants.^25^

The CD59 preproprotein^26^ of 128 amino acids contains a 25 amino acids‐long N‐terminal signal peptide, which is crucial for the protein's translocation to the endoplasmic reticulum (ER). A 26 amino acids‐long C‐terminal signal peptide facilitates the protein's attachment to the glycosylphosphatidylinositol (GPI) moiety.^27^, ^28^ Upon translocation of the CD59 preproprotein into the ER lumen, the N‐terminal signal peptide is removed, generating the CD59 proprotein.^29^, ^30^ The C‐terminal signal peptide is recognized by the GPI transamidase, which cleaves and replaces it with a preassembled GPI through transamidation,^31^ generating the mature GPI‐anchored CD59.^29^, ^30^ Hence, the mature CD59 glycoprotein is 77 amino acids long^32^, ^33^, ^34^, ^35^, ^36^, ^37^, ^38^, ^39^ and exposed in its entirety on the cell's surface.

The human *CD59* gene (MIM#107271) is located on chromosome 11 (11p13) and encodes the CD59 preproprotein.^26^ The 40.4 kb long gene (NG_008057.1) consists of 6 exons. The first 3 exons are non‐coding, while the preproprotein (NM_203330.2) is encoded by the nucleotide sequences in exons 4–6. The 19th nucleotide in exon 4 represents the “A” of the start codon (ATG) of the coding sequence.

The first case of CD59 loss on erythrocytes was observed in a 20‐year‐old paroxysmal nocturnal hemoglobinuria patient in Japan in 1990.^19^, ^40^ Molecular analysis revealed 2 single‐base deletions at nucleotide positions 123 and 361, resulting in frameshift variations at the N‐terminal region of the CD59 protein (p.Val42fs and p.Ala121fs).^20^ Since then, 6 additional pathogenic variants have been identified and published (c.67 + 1C>A, p.Tyr29Asp, p.Asp49Val, p.Asp49fs, p.Cys89Tyr, p.Ser108Ter).^13^, ^14^, ^15^, ^16^, ^21^, ^22^, ^23^, ^24^, ^25^ Furthermore, 13 non‐synonymous variants, 3 frameshift variants and 1 nonsense variant have been listed in the ClinVar database.^41^

Recent large‐scale whole‐genome and whole‐exome sequencing projects, such as All of Us (AoU),^42^ The IndiGenomes database (IndiGen),^43^ The GenomeAsia 100K Project (GA100K),^44^ the 1000 Genomes Project (1000GP),^45^ the South Asian Genomes and Exomes database (SAGE),^46^ ClinVar,^47^ The International HapMap Project,^48^ The Human Genome Diversity Project (HGDP),^49^, ^50^ The NHS England 100,000 Genomes Project,^51^ the Three Million African Genomes (3MAG),^52^ and the UK Biobank project,^53^ are generating vast datasets of human variation across globally diverse and genetically heterogeneous populations. Using these genome databases, the genetic variability among populations can be systematically evaluated.^54^ Worldwide differences in the prevalence and distribution of genetic variants can influence many diseases at the population level.^55^ Understanding global genetic diversity can provide insights into the mechanisms underlying disease, risk assessment, diagnostic and therapeutic strategies, and public health decision‐making models.^55^, ^56^

The PredictSNP^57^ metaserver is a consensus classifier that combines 6 prediction methods to provide accurate and robust predictions regarding the effects of amino acid substitutions on protein function. These predictions are based on the evolutionary, physico‐chemical, and structural characteristics of a specific substitution. The predictions are further supported by experimental annotations from the Protein Mutant Database^58^ and UniProt database.^59^ PredictSNP demonstrated its highest accuracy for nucleotide sites located in highly conserved regions of a protein, but may be less accurate for non‐conserved regions.^60^

Variants in the *CD59* gene have not been systematically collated and evaluated for pathophysiologic effects. We aimed at a comprehensive analysis of *CD59* gene variants from real‐world data in publicly available genome databases of large populations. We explored if human‐based computational models could distinguish neutral variants from deleterious ones. Such *CD59* alleles could be prioritized for red cell genotyping, experimental characterization, and pathological investigation.

## 2 MATERIALS AND METHODS

### 2.1 Data mining of *CD59* variants

Human *CD59* genomic (NG_008057.1), mRNA (NM_203330.2) and protein (NP_976075.1) sequences were obtained from the National Center for Biotechnology Information (NCBI). *CD59* variants in the coding sequence, found in exons 4, 5, and 6, and the +1 and +2 positions of the donor and acceptor splice sites were identified from published manuscripts using the PubMed database. When available, their functional impacts were also extracted from published literature.

We excluded the first 3 *CD59* exons from final data analysis, as these exons are non‐coding and absent in whole‐exome sequencing databases^61^ or published literature. These exons are also presumed to be non‐deleterious to CD59 protein structure and function. Whole‐genome and whole‐exome datasets, including All of Us (AoU),^42^ The IndiGenomes database (IndiGen),^43^ The GenomeAsia 100 K Project (GA100K),^44^ and the 1000 Genomes Project (1000GP),^45^ were systematically screened. Additionally, the South Asian Genomes and Exomes database (SAGE),^46^ which includes data from 6 datasets of South Asian populations, was considered.^45^, ^62^, ^63^, ^64^, ^65^, ^66^ Our search in public databases was supplemented by reviewing reference lists from identified original research and review articles for potentially relevant reports. The ClinVar^47^ database was also searched for pathogenic *CD59* variants (accessed Feb 25, 2025). All *CD59* variants in the databases were collated, and no filtering restrictions were applied.

### 2.2 Computational modeling of *CD59* variants

The PredictSNP^57^ metaserver (accessed Feb 25, 2025) was used to determine a consensus prediction of the functional impact of non‐synonymous single nucleotide variants (SNVs). The accuracy of the *in silico* prediction for non‐synonymous SNVs was validated by comparing the predicted effect with clinically reported outcomes in published studies.

To investigate the similarity between the CD59 protein in humans (NP_976075.1), chimpanzees (JAA32635.1) and mice (NP_001403853.1), a multiple sequence alignment was performed using Clustal Omega with default settings.^67^

### 2.3 Statistical description

Fisher's exact or the Chi‐square test was performed to assess differences in the distribution of categorical variables. The Kolmogorov–Smirnov test^68^ was applied to determine the distribution of non‐synonymous nucleotide variants across the *CD59* coding sequence. *p* < .05 was considered statistically significant.

## 3 RESULTS

Data were compiled from 6 online databases^42^, ^43^, ^44^, ^45^, ^46^ to describe the genetic variability of the *CD59* gene for 488,592 individuals. We analyzed 387 nucleotides of the *CD59* coding sequence (CDS) and the +1 and +2 positions of the donor and acceptor splice sites, for a total of 391 nucleotides. In total, 6881 individuals (0.7%) harbored variant alleles (Table 1). The prevalence of variants differed significantly among the 4 major populations analyzed.

**TABLE 1:** *CD59* variant frequency in 6 genome databases.

Population	Total individuals	Variant alleles	
		*n* ^a^	Frequency
African	85,081	3794	2.2%
Latin American	78,740	773	0.5%
White	257,310	1844	0.4%
Asian	20,261	55	0.1%
Unknown	47,200	415	0.4%
Total	488,592	6881	0.7%

^a^ Statistically significant difference by chi‐square test, two‐sided, *p* <.00001.

### 3.1 *CD59* variants

These individuals carried 160 distinct variants (Table 2). Among the variants, 147 were SNVs (91.2%), including 93 non‐synonymous variants, 53 synonymous variants, and 1 nonsense variant (Tables S1 and S2). The 93 non‐synonymous variants resulted in 92 amino acid changes, as both c.286A>G and c.288A>C lead to p.Phe96Leu. Additionally, there were 6 single nucleotide deletions (3.9%), 1 in‐frame deletion (0.7%), and 3 single nucleotide splice site variants (1.9%) across the analyzed sequence (Table S1). Among the 22 variants with 10 or more observed alleles (Table 3), 12 were synonymous, with 8 occurring in at least 3 major populations.

**TABLE 2:** *CD59* variants position and effect.

*CD59* exon	Coding sequence length (bp)			Observed variant type (*n*)					
		Coding sequence (CDS)				Deletion/duplication			
		Non‐Synonymous	Synonymous	Nonsense	Splice site	With frameshift	Without frameshift	Total	*p* ^a^
4	67	24	9	0	1	0	0	34	.415
5	102	24	16	1	2	3	0	45	
6	218	45	28	1	0	5	1	81	
Total	387	93	53	2	3	8	1	160	

Abbreviation: bp, base pairs.

^a^ Chi‐square test between total number of coding nucleotides (column 2) in each exon and the total number of observed variants (column 9), 3 × 2 contingency table, two‐sided.

**TABLE 3:** *CD59* variants with 10 or more observations among 488,592 individuals.

Position and substitution		PredictSNP classification	Observations					
Nucleotide	Amino acid		*n*	Population^a^				
				Af	W	A	L	U
c.126G>C	p.Val42=	NA	2311	x	x	x	x	x
c.18C>T	p.Gly6=	NA	1243	x	x	x	x	x
c.222G>A	p.Asp74=	NA	916	x	x		x	x
c.54G>A	p.Val18=	NA	676	x	x	x	x	x
c.150C>T	p.Ala50=	NA	621	x	x	x	x	x
c.72A>G	p.His24=	NA	211	x	x		x	x
c.30G>A	p.Phe10=	NA	179	x	x		x	x
c.171C>T	p.Gly57=	NA	86	x	x			x
c.335_341delGAA	p.Leu115del	NA	49	x			x	x
c.71 T>C	p.His24Arg	Neutral	46	x	x	x	x	x
c.52C>T	p.Val18Ile	Neutral	35				x	x
c.98 T>G	p.Asn33Thr	Neutral	32	x	x		x	
c.382G>A	p.Pro128Ser	Deleterious	31		x		x	x
c.149G>A	p.Ala50Val	Neutral	25		x	x	x	x
c.291G>A	p.Asn97=	NA	18	x	x	x	x	x
c.299A>C	p.Leu100Arg	Deleterious	17		x			x
c.266C>T	p.Cys89Tyr	Deleterious	15	x	x			
c.124C>T	p.Val42Ile	Neutral	15	x	x	x	x	x
c.288A>G	p.Phe96=	NA	11	x	x			
c.313 T>G	p.Thr105Pro	Deleterious	11		x	x		
c.198C>T	p.Lys66=	NA	11	x	x			
c.219G>A	p.Asn73=	NA	10	x	x			x

*Note*: NA, not applicable, because a synonymous nucleotide variant does not affect the protein sequence.

^a^ Af = African, W=White, A = Asian, L = Latin American, U = Unknown.

**FIGURE 1:**
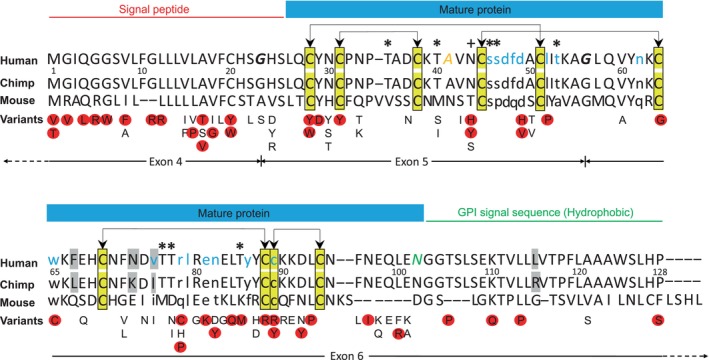
CD59 protein sequence analyzed in the study. The CD59 preproprotein has 128 amino acids, encoded by nucleotide sequences straddling 3 exons (exon 4, 5, and 6). The mature CD59 protein (blue line) consists of 77 amino acids and is generated by the removal of the 25 amino acids‐long signal peptide at the amino‐terminal (red line) and the 26 amino acids‐long hydrophobic GPI signal sequence at the carboxy‐terminal end (green line).^36^, ^37^ All 10 cysteine residues in the mature CD59 protein form disulfide bonds (black arrows).^36^ Sixteen residues are involved in the MAC inhibitory function (lowercase, blue) while 1 residue each is involved in the transportation of CD59 onto the cell surface (A, orange italics) and its attachment to GPI anchor (N, green italics).^36^ Eight residues enable the O‐glycosylation (*)^39^ and 1 residue the N‐glycosylation (+).^38^ The codons of 2 amino acids straddle either of the 2 exon boundaries (bold italics). Amino acid sequences of human (NP_976075.1), chimpanzee (JAA32635.1) and mouse (NP_001403853.1) are aligned, and the amino acid differences between human and chimpanzee are indicated (gray). The PredictSNP categorized 92 variants (amino acid changes) deleterious (red blots) or neutral (white). [Color figure can be viewed at wileyonlinelibrary.com]

When analyzing sequence alignments, we identified 4 differences between the human and chimpanzee sequences, as well as 84 differences between the human and mouse sequences (Figure 1). Blotting the variants observed in humans, the distribution seemed to differ among the 3 CD59 preproprotein segments comprising signal peptide, mature protein, and GPI signal sequence.

### 3.2 Distribution of amino acid substitutions

The 160 variants were equally distributed among the 3 exons, including +1 and +2 positions of the donor and acceptor splice sites (*p* = .415, Table 2). However, the observed distribution deviated from a normal distribution (Kolmogorov–Smirnov test) for the 93 non‐synonymous SNVs across the 387 nucleotides in the *CD59* coding sequence (*p* = .00005, data not shown) and the 231 nucleotides in the CD59 mature protein (*p* = .00031, data not shown).

We compared the amino acid variants and individuals observed in each of the 3 protein segments (Table S3). The number of amino acid variants did not significantly differ between the signal peptide and mature protein (*p* = .4131). Among the individuals with variants, the numbers differed significantly, with the GPI signal sequence having the least and the signal peptide the highest rates (Table S3).

### 3.3 *CD59* variants in patients

Pathologic effects in patients were reported for 35 variants (Table S4); 8 of these were published,^13^, ^14^, ^15^, ^16^, ^18^, ^19^, ^20^, ^21^, ^22^, ^23^, ^24^, ^25^ and 2 of the published variants (p.Tyr29Asp^18^ and p.Cys89Tyr^13^, ^14^) were also documented in ClinVar (Table S4). Due to incomplete pathology reports, 22 variants documented in ClinVar (Table S4) currently lack an associated clinical description. Only 3 published variants (p.Asp49Val, p.Cys89Tyr, p.Ser108Ter)^13^, ^14^, ^15^, ^16^, ^25^ were independently observed in the genome databases (Table S1) as single observations (heterozygotes).

### 3.4 Correlation of clinical data with protein structure predictions

Among the 92 amino acid changes, PredictSNP classified 43 as deleterious and 49 as neutral (Table S1). Notably, the 3 non‐synonymous *CD59* variants reported in patients (p.Tyr29Asp, p.Asp49Val, and p.Cys89Tyr)^13^, ^14^, ^15^, ^16^, ^18^, ^21^, ^22^, ^23^ (Table S4) were indeed classified as “deleterious” by the PredictSNP algorithm, with accuracies reaching 87% (Table S1). The expected accuracies for the remaining 40 variants reported as deleterious ranged from 51% to 97%, with a median of 76% (Table S5). Some of these 40 variants were located in functionally and structurally important amino acid locations in the CD59 protein.^32^, ^69^ Among the 13 non‐synonymous variants present in the ClinVar database (Table S4), the computational modeling predicted 8 to be deleterious and 5 to be neutral, all with high^70^ expected accuracies (Table S1). Only 5 of these ClinVar variants were associated with primary CD59 deficiency (p.Met1Thr, p.Cys28Tyr, p.Tyr29Asp, p.Cys64Gly, and p.Cys89Tyr; Table S4). Among the 80 CD59 amino acid substitutions present only in the genome databases, 35 were classified as deleterious and 45 as neutral (Table 4).

**TABLE 4:** PredictSNP predictions for CD59 amino acid substitutions.

PredictSNP classification	Variants^a^ (*n*)			Total
	Genome databases only	ClinVar only	Genome database and ClinVar	
Deleterious	35	5	3	43
Neutral	45	4	1	49^b^
Total	80	9	4	92^b^

^a^ See Table S1 for variants.

^b^ Two nucleotide variants encoded the same amino acid substitution (p.Phe96Leu).

### 3.5 Population frequency of *CD59* variants

Across all 4 large populations, the c.126G>C (p.Val42=) variant was the most frequent, with an overall allele frequency of 0.47% (Table 3). Among non‐synonymous variants, the most common variant classified as deleterious by PredictSNP was c.382G>A (p.Pro128Ser), observed in Whites (0.009%) and Latin Americans (0.005%) (Table S1). Collectively, across all populations, approximately 1 in 71 individuals was found to be a carrier of any variant located in *CD59*, regardless of pathogenicity (Table 5). However, the prevalence of individuals with the highest risk of harboring a deleterious non‐synonymous *CD59* variant in homozygous or compound heterozygous form was markedly low across all populations analyzed (Table 5). Even after accounting for the average inbreeding coefficient in Whites (0.00116),^71^ the probability of observing a White individual harboring homozygous deleterious alleles^71^, ^72^ was rare, ranging from 1 in 4,477,526 to 1 in 110,539,570 (Table S6).

**TABLE 5:** Estimated prevalence of any *CD59* variant in different populations.

Population	Estimated prevalence of an individual in the population^a^				
	Any variant allele		Deleterious allele		
			Heterozygous		Homozygous or compound heterozygous for any deleterious allele
	Heterozygous^b^	Homozygous or compound heterozygous^c^	Any	Most common^d^	
African	1 in 22	1 in 242	1 in 7090	1 in 17,016	1 in 25,134,050
Latin American	1 in 102	1 in 5202	1 in 4921	1 in 19,685	1 in 12,108,120
White	1 in 139	1 in 9660	1 in 3386	1 in 11,187	1 in 5,732,498
Asian	1 in 368	1 in 67,712	1 in 2533	1 in 5065	1 in 3,208,044
All populations	1 in 71	1 in 2520	1 in 3908	Not applicable	1 in 7,636,232

^a^ See Tables S1 and S3 for *CD59* variants.

^b^ “Heterozygous” refers to a genotype comprising the common allele, such as the *CD59* NG_008057.1 allele, and a variant allele.

^c^ “Compound heterozygous” refers to a genotype comprising 2 different variant alleles at a particular gene, such as *CD59*, one distinct variant allele on each chromosome and not the common wild type (reference) allele, such as the *CD59* NG_008057.1 allele.^12^

^d^ The most common deleterious allele is c.11T>A for the African population (c.382G>A for White and Latin American, and c.313T>G for Asian).

## 4 DISCUSSION

Proactively identifying deleterious variants through whole genome sequencing^73^ will help expand the repertoire of confirmed pathogenic variants, especially when coupled with follow‐up monitoring of individuals harboring these variants for clinical symptoms. This data can support cascade screening^74^ to identify family members at risk or with latent disease and inform those families with the majority of variants that pose no clinical risk. The National Heart, Lung, and Blood Institute (NHLBI) working group of 2010^75^ and other institutions emphasize the responsibility to return genetic results promptly, especially when therapeutic or preventive interventions are available.^76^

A complete absence of complement‐regulatory CD59 causes recurrent ischemic strokes,^14^, ^15^ neuropathy,^13^, ^15^, ^16^, ^18^, ^22^, ^23^ and chronic hemolysis.^13^, ^15^, ^19^, ^20^, ^21^ Reduced CD59 expression has also been linked to neurodegeneration in Alzheimer's disease,^77^ bronchiolitis obliterans syndrome,^78^ and neuromyelitis optica spectrum disorder.^79^ We compiled 160 distinct nucleotide variants of *CD59* from 488,592 individuals across global genome databases, including 14 known pathogenic variants and 37 potentially deleterious variants, as assessed using the *in silico* PredictSNP metaserver (Tables S4 and S5).

The vast majority of homozygous *CD59* pathogenic variants identified so far have been largely restricted within families.^13^, ^14^, ^15^, ^16^, ^21^, ^22^, ^23^ We found that potentially deleterious variants in the *CD59* gene are extremely rare in the general population. Even after incorporating the known inbreeding coefficient in the White population (0.00116),^71^, ^80^ the estimated prevalence of an individual in the population for the most common deleterious variant c.382G>A was 1 in 4.5 million (Table S6). The inbreeding coefficient, ranging from 0 to 1, represents the probability of inheriting an identical haplotype from both parents at an autosomal locus.^81^ A higher inbreeding coefficient, observed in countries in the Middle East (~0.024), North Africa (~0.03), and parts of Western Asia (~0.033), indicates a greater degree of consanguinity.^80^ Our finding is consistent with the association between pathogenic *CD59* variants and serious illnesses. A similar pattern is expected for the *ACHE* gene, which encodes the Cartwright blood group system, as individuals lacking the ACHE protein have never been observed, highlighting its vital role in fetal development.^82^, ^83^ This contrasts with other rare null phenotypes of blood group systems, such as the Bombay phenotype in Europe, which results from diverse, sporadic, nonfunctional alleles.^71^

In addition to the direct pathogenic effects, *CD59* variants may also contribute to previously unexplained cases of infusion‐related hemolysis, such as those occurring during ABO‐incompatible platelet transfusions. While these transfusions are routinely performed and generally regarded as safe—especially with the use of strategies^84^, ^85^ to mitigate the risk of hemolysis from ABO‐incompatible platelets—some instances of unexplained hemolysis might be linked to reduced CD59 activity, potentially caused by deleterious *CD59* variants.

Using predictive models, the haploinsufficiency score of *CD59* is 97.5,^86^ and the probability of being loss‐of‐function (LoF) intolerant (pLI) is 0.01.^87^ According to these data, heterozygous missense variants are likely sufficient to reduce the CD59 protein expression or function, or both. In addition to CD59, variants in other membrane‐bound complement regulatory proteins,^88^ such as CD35, CD46, and CD55, as well as immune response factors, such as titers of antibody^89^, ^90^ and specific HLA haplotypes,^91^, ^92^, ^93^, ^94^, ^95^ may contribute to hemolysis.

PredictSNP classified 43 of the non‐synonymous *CD59* variants as deleterious, 3 of which were indeed observed in patients.^13^, ^14^, ^15^, ^16^, ^18^, ^21^, ^22^, ^23^ The list was largely based on the known or potential effects of the variant on CD59 protein structure or function.^32^, ^69^ Except for 2 variants, c.127T>G and c.127T>A, established to have no effect on the MAC inhibitory function of the CD59 protein,^69^ the most common non‐synonymous deleterious variant c.382G>A and the other PredictSNP‐classified deleterious variants could be pathogenic. We cannot exclude that some *CD59* variants predicted as neutral affect the MAC assembly without altering the CD59 protein's structure, which could make them pathogenic.

The amino acid variants in the *CD59* gene were unevenly distributed (Figure 1), based on a deviation from a normal distribution of non‐synonymous SNVs across the *CD59* coding sequence, and a mature sequence was observed (Table S3). Significant differences were also observed between the signal peptide and GPI signal sequence, suggesting the GPI signal sequence is highly conserved, as it is critical for red cell survival. These findings for CD59 can be matched in the future with those of the other 6 GPI‐linked blood group systems (Cartwright, Dombrock, Cromer, JohnMiltonHagen, Kanno, EMM), as well as with other GPI‐linked proteins found on the surface of various human cell types, some of which may be recognized as blood group systems in the future.

The 5 non‐synonymous variants listed in ClinVar: c.233C>T (p.Arg78His), c.288A>C (p.Phe96Leu), c.292C>G (p.Glu98Gln), c.302T>G (p.Glu101Ala), and c.361C>A (p.Ala121Ser) were computationally predicted to be neutral (Table S4). However, due to the lack of any associated clinical conditions for these variants, we cannot exclude that such variants could still be pathogenic. To functionally annotate *CD59* variants, several study approaches are feasible. First, patients who experience red cell hemolysis after transfusions, despite no changes in commonly suspected blood group antigens,^96^, ^97^ could be tested for the presence of *CD59* variants. Second, prospective follow‐up studies could be conducted on transfused patients carrying CD59 variants to assess the occurrence of red cell hemolysis. Third, investigating individuals who have received blood from donors with *CD59* variants may provide additional insights. Finally, *CD59* variants with an allele frequency greater than 1% in the general population may imply their non‐pathogenic nature.^98^

The databases used in this study were generated using different sequencing technologies, coverage depths, and variant‐calling pipelines, affecting the consistency and reliability of variant detection. Especially rare variants, observed only once, may represent technical artifacts. Such methodological details should be considered when interpreting the data. In addition, *in silico* prediction tools, while useful, have notable limitations and often misclassify variants due to overreliance on conservation.^99^, ^100^ Especially variants located in regions with low evolutionary conservation or outside well‐characterized functional domains may lack sufficient evidence. Hence, computational predictions should be interpreted cautiously and, when possible, corroborated or validated by experimental confirmation.

Systematic collation and analysis of variants in global genome databases is instrumental for identifying disease‐susceptible variants, with machine learning potentially aiding in predicting their functional impacts. Our study provides a comprehensive report on *CD59* variants, advancing our understanding across populations, and enabling novel diagnostic approaches in clinical care.

## AUTHOR CONTRIBUTIONS

WAF and KS conceived the study. TR and KS screened the databases and compiled the variants. WAF and KS analyzed and discussed the data. WAF and KS wrote drafts and WAF the final manuscript.

## FUNDING INFORMATION

This work was supported by the Intramural Research Program (projects ZIC CL002128 and RASCL 727301) of the NIH Clinical Center at the National Institutes of Health.

## CONFLICT OF INTEREST STATEMENT

The authors declared having no competing financial interest.

## Supporting information


**Data S1:** Supporting information
