# Supplementary material for: Genetic variants in the CD59 gene: An exploratory study of large genome databases
Source: Transfusion. 2025 Jul 30;65(9):1682–92. doi: 10.1111/trf.18331 (PMC12432816; doi:10.1111/trf.18331)
Supplement: Supplementary file 1 — Data S1: Supporting information [file TRF-65-1682-s001.pdf]

Table S1. *CD59* variants retrieved from different databases

		Substitution			Observations			Computational analysis results	
Location	Variant number	Reference number	Nucleotide†	Amino acid‡	Alleles observed	Population§	Reference	PredictSNP Classification	Expected accuracy (%)‖
Exon 4	1	rs368463258	c.1T>C	p.Met1Val	1	African	AoU	Deleterious	51
					3	White	AoU		
					3	Latin American	AoU		
	2	VCV003599568.1	c.2A>G	p.Met1Thr	n.a.	n.a.	ClinVar	Deleterious	51
	3	rs767728205	c.5C>A	p.Gly2Val	1	Unknown	AoU	Deleterious	76
	4	rs759648723	c.11T>A	p.Gln4Leu	5	African	AoU	Deleterious	51
	5	rs750381382	c.13C>T	p.Gly5Arg	1	White	AoU	Deleterious	87
	6	n.a.	c.16C>A	p.Gly6Trp	1	White	AoU	Deleterious	76
	7	rs111771149	c.18C>T	p.Gly6=	83	African	AoU	n.p.	n.p.
					923	White	AoU		
					148	Latin American	AoU		
					78	Unknown	AoU		
					1	African	1000GP		
					1	Latin American	1000GP		
					5	White	1000GP		
					2	Asian	SAGE		
					1	African	GA100K		
					1	White	GA100K		
	8	rs111771149	c.18C>A	p.Gly6=	3	White	AoU	n.p.	n.p.
	9	rs761729220	c.22C>A	p.Val8Phe	1	African	AoU	Deleterious	65
					5	White	AoU		
	10	rs377286139	c.23A>G	p.Val8Ala	1	White	AoU	Neutral	63
	11	rs143169907	c.30G>A	p.Phe10=	24	African	AoU	n.p.	n.p.
					127	White	AoU		
					17	Latin American	AoU		
					9	Unknown	AoU		
					2	White	1000GP		
	12	rs377227068	c.31C>T	p.Gly11Arg	7	White	AoU	Deleterious	72
	13	VCV002827178.1	c.33C>G	p.Gly11=	n.a.	n.a.	ClinVar	n.p.	n.p.
	14	n.a.	c.35A>C	p.Leu12Arg	1	White	AoU	Deleterious	87
	15	rs1406884887	c.37G>A	p.Leu13=	1	Latin American	AoU	n.p.	n.p.
	16	rs372568051	c.42G>A	p.Leu14=	1	White	AoU	n.p.	n.p.

					2	Latin American	AoU		
	17	rs971343343	c.43C>T	p.Val15Ile	1	African	AoU	Neutral	60
					6	White	AoU		
					1	Latin American	AoU		
					1	Unknown	AoU		
	18	rs971343343	c.43C>A	p.Val15Phe	1	Asian	AoU	Neutral	83
	19	n.a.	c.46G>C	p.Leu16Val	1	Latin American	AoU	Neutral	60
	20	rs781460834	c.46G>A	p.Leu16=	1	African	AoU	n.p.	n.p.
					5	White	AoU		
					1	Unknown	AoU		
	21	rs1229617535	c.47A>G	p.Leu16Pro	1	Latin American	AoU	Deleterious	97
	22	n.a.	c.49C>T	p.Ala17Thr	1	Asian	AoU	Deleterious	60
	23	rs1291876268	c.49C>A	p.Ala17Ser	1	White	AoU	Neutral	87
	24	rs1447510141	c.50G>A	p.Ala17Val	1	White	AoU	Deleterious	51
	25	rs368548962	c.51A>G	p.Ala17=	3	White	AoU	n.p.	n.p.
					6	Latin American	AoU		
	26	rs745311147	c.52C>T	p.Val18Ile	34	Latin American	AoU	Neutral	60
					1	Unknown	AoU		
	27	rs1564975530	c.53A>C	p.Val18Gly	2	Latin American	AoU	Deleterious	87
	28	rs2231456	c.54G>A	p.Val18=	490	African	AoU	n.p.	n.p.
					25	White	AoU		
					91	Latin American	AoU		
					5	White	AoU		
					51	Unknown	AoU		
					11	African	1000GP		
					1	Asian	SAGE		
					2	African	GA100K		
	29	n.a.	c.55A>G	p.Phe19Leu	1	Unknown	AoU	Neutral	75
	30	n.a.	c.59C>T	p.Cys20Tyr	1	Asian	AoU	Deleterious	61
	31	n.a.	c.60G>C	p.Cys20Trp	1	Unknown	AoU	Deleterious	87
	32	rs146915059	c.65G>A	p.Ser22Leu	6	White	AoU	Neutral	75
	33	n.a.	c.67C>T	p.Gly23Ser	2	Asian	GA100K	Neutral	63
Intron 4	34	n.a.	c.67+1C>A	n.p.	2	White	[1]	n.p.	n.p.
	35	rs1853851098	c.68-2T>C	n.p.	2	African	AoU	n.p.	n.p.
Exon 5	36	rs370362902	c.70G>C	p.His24Asp	4	White	AoU	Neutral	83
	37	rs370362902	c.70G>A	p.His24Tyr	1	White	AoU	Neutral	60
					2	Latin American	AoU		
	38	rs560254666	c.71T>C	p.His24Arg	7	African	AoU	Neutral	60
					1	Asian	AoU		

				26	White	AoU		
				2	Latin American	AoU		
				8	Unknown	AoU		
				2	African	1000GP		
39	rs2231457	c.72A>G	p.His24=	5	African	AoU	n.p.	n.p.
				91	White	AoU		
				103	Latin American	AoU		
				11	Unknown	AoU		
				1	African	1000GP		
40	n.a.	c.76G>A	p.Leu26=	2	African	1000GP	n.p.	n.p.
41	rs1853849281	c.83C>T	p.Cys28Tyr	1	Unknown	AoU	Deleterious	87
	VCV001685256.1			n.a.	n.a.	ClinVar		
42	rs1853849042	c.84G>C	p.Cys28Trp	1	African	AoU	Deleterious	87
43	rs1564972905	c.85A>C	p.Tyr29Asp	2	White	[2]	Deleterious	87
	VCV000634643.3			n.a.	Asian	ClinVar		
44	n.a.	c.88T>A	p.Asn30Tyr	1	Unknown	AoU	Neutral	60
45	rs769967114	c.89T>C	p.Asn30Ser	1	African	AoU	Neutral	83
				1	Asian	AoU		
46	n.a.	c.89T>G	p.Asn30Thr	1	Asian	IndiGen	Neutral	83
47	rs1853848553	c.92C>T	p.Cys31Tyr	1	White	AoU	Deleterious	87
48	rs755154716	c.98T>G	p.Asn33Thr	2	African	AoU	Neutral	83
				27	White	AoU		
				3	Latin American	AoU		
49	rs747156478	c.99G>C	p.Asn33Lys	1	White	AoU	Neutral	65
				4	Latin American	AoU		
50	rs747156478	c.99G>A	p.Asn33=	1	African	AoU	n.p.	n.p.
				4	White	AoU		
				2	Latin American	AoU		
				1	Asian	IndiGen		
51	n.a.	c.109C>T	p.Asp37Asn	2	African	AoU	Neutral	83
52	n.a.	c.118T>A	p.Thr40Ser	1	Asian	AoU	Neutral	83
53	rs370913821	c.119G>A	p.Thr40Ile	9	African	AoU	Neutral	60
54	VCV002811866.1	c.120T>A	p.Thr40=	n.a.	n.a.	ClinVar	n.p.	n.p.
55	rs577673753	c.123G>A	p.Ala41=	5	African	AoU	n.p.	n.p.
				1	White	AoU		
				1	African	1000GP		
56	rs2133545024	c.123delG	p.Val42fs	1	Asian	[3,4]	n.p.	n.p.
57	rs763958932	c.124C>T	p.Val42Ile	1	African	AoU	Neutral	83
				4	White	AoU		
				6	Latin American	AoU		

				1	Asian	AoU		
				3	Unknown	AoU		
58	rs2231458	c.126G>C	p.Val42=	1966	African	AoU	n.p.	n.p.
				12	White	AoU		
				163	Latin American	AoU		
				129	Unknown	AoU		
				29	African	1000GP		
				3	Asian	SAGE		
				9	African	GA100K		
59	n.a.	c.127T>G	p.Asn43His	1	White	AoU	Deleterious	72
60	n.a.	c.127T>A	p.Asn43Tyr	1	African	AoU	Deleterious	51
61	rs1351382260	c.128T>C	p.Asn43Ser	1	Latin American	AoU	Neutral	74
62	rs147726303	c.129A>G	p.Asn43=	5	White	AoU	n.p.	n.p.
				1	Unknown	AoU		
63	n.a.	c.132A>G	p.Cys44=	1	White	AoU	n.p.	n.p.
64	n.a.	c.135T>A	p.Ser45=	3	Latin American	AoU	n.p.	n.p.
65	VCV003599567.1	c.143_144delAA	p.Phe48Ter	n.a.	n.a.	ClinVar	n.p.	n.p.
66	n.a.	c.145C>G	p.Asp49His	3	African	AoU	Deleterious	87
67	n.a.	c.146T>A	p.Asp49Val	1	Unknown	AoU	Deleterious	87
				8	White	[5,6]		
68	rs587777149	c.146delT	p.Asp49fs	6	White	[7-9]	n.p.	n.p.
69	n.a.	c.148C>T	p.Ala50Thr	4	African	AoU	Neutral	83
70	rs759088791	c.149G>A	p.Ala50Val	11	White	AoU	Neutral	75
				11	Latin American	AoU		
				1	Asian	AoU		
				2	Unknown	AoU		
71	rs149071679	c.150C>T	p.Ala50=	99	African	AoU	n.p.	n.p.
				5	Asian	AoU		
				338	White	AoU		
				133	Latin American	AoU		
				36	Unknown	AoU		
				2	African	1000GP		
				4	Latin American	1000GP		
				1	White	1000GP		
				2	Asian	GA100K		
				1	White	GA100K		
72	rs1853844038	c.153A>G	p.Cys51=	1	White	AoU	n.p.	n.p.
73	n.a.	c.155A>G	p.Leu52Pro	1	White	AoU	Deleterious	87
74	n.a.	c.156G>C	p.Leu52=	5	White	AoU	n.p.	n.p.

	75	rs533392692	c.156G>A	p.Leu52=	3	Asian	AoU	n.p.	n.p.
					3	Asian	1000GP		
					1	Asian	GA100K		
					1	Asian	IndiGen		
	76	n.a.	c.162G>C	p.Thr54=	1	White	AoU	n.p.	n.p.
	77	VCV002750919.1	c.165T>C	p.Lys55=	n.a.	n.a.	ClinVar	n.p.	n.p.
	78	rs1457484403	c.168A>G	p.Ala56=	1	African	AoU	n.p.	n.p.
Intron 5	79	n.a.	c.169+1C>G	n.p.	1	Asian	AoU	n.p.	n.p.
					2	Latin American	AoU		
Exon 6	80	rs2231459	c.171C>T	p.Gly57=	62	African	AoU	n.p.	n.p.
					19	White	AoU		
					4	Unknown	AoU		
					1	African	1000GP		
	81	rs2231459	c.171C>A	p.Gly57=	1	Latin American	AoU	n.p.	n.p.
	82	rs762390640	c.179A>G	p.Val60Ala	7	White	AoU	Neutral	60
	83	rs1853488449	c.180C>T	p.Val60=	1	Latin American	AoU	n.p.	n.p.
	84	VCV003027278.9	c.183_184dup	p.Asn62fs	n.a.	n.a.	ClinVar	n.p.	n.p.
	85	rs777232599	c.189C>T	p.Lys63=	1	Latin American	AoU	n.p.	n.p.
	86	rs1554939509 VCV000522723.4	c.190A>C	p.Cys64Gly	n.a.	n.a.	ClinVar	Deleterious	87
	87	rs961953611	c.190delA	p.Cys64fs	3	African	AoU	n.p.	n.p.
	88	n.a.	c.192A>G	p.Cys64=	1	Asian	AoU	n.p.	n.p.
	89	n.a.	c.195C>G	p.Trp65Cys	1	Unknown	AoU	Deleterious	87
	90	rs1294522605	c.198C>T	p.Lys66=	4	African	AoU	n.p.	n.p.
					7	White	AoU		
	91	rs1213555091	c.202C>G	p.Glu68Gln	1	White	AoU	Neutral	65
	92	n.a.	c.204C>T	p.Glu68=	1	African	AoU	n.p.	n.p.
	93	rs768902780	c.214A>C	p.Phe72Val	1	White	AoU	Neutral	63
	94	n.a.	c.216G>C	p.Phe72Leu	1	White	AoU	Neutral	63
	95	rs761038612	c.219G>A	p.Asn73=	1	African	AoU	n.p.	n.p.
					8	White	AoU		
					1	Unknown	AoU		
	96	rs144931418	c.220C>T	p.Asp74Asn	2	African	AoU	Neutral	83
					2	White	AoU		
					2	Latin American	AoU		
	97	rs2231460	c.222G>A	p.Asp74=	828	African	AoU	n.p.	n.p.
					7	White	AoU		
					2	Latin American	AoU		
					57	Unknown	AoU		

				18	African	1000GP		
				4	African	GA100K		
98	rs745985153	c.223C>T	p.Val75Ile	1	African	AoU	Neutral	83
				2	White	AoU		
				1	Latin American	AoU		
				1	Unknown	AoU		
99	n.a.	c.230G>T	p.Thr77Asn	1	African	AoU	Neutral	74
100	rs1853485378	c.230G>A	p.Thr77Ile	2	White	AoU	Neutral	83
101	VCV002866244.1	c.231G>A	p.Thr77=	n.a.	n.a.	ClinVar	n.p.	n.p.
102	rs146075291	c.232G>A	p.Arg78Cys	1	White	AoU	Deleterious	76
				1	Latin American	AoU		
				1	White	1000GP		
103	rs546769632	c.233C>T	p.Arg78His	2	White	AoU	Neutral	83
				n.a.	n.a.	ClinVar		
	VCV003616832.1			1	Latin American	AoU		
104	rs546769632<br>VCV001026000.4	c.233C>G	p.Arg78Pro	n.a.	n.a.	ClinVar	Deleterious	61
105	VCV002766804.1	c.235A>G	p.Leu79=	n.a.	n.a.	ClinVar	n.p.	n.p.
106	n.a.	c.238T>C	p.Arg80Gly	n.a.	Asian	[10]	Neutral	83
107	n.a.	c.241C>T	p.Glu81Lys	1	White	AoU	Deleterious	87
108	n.a.	c.243T>C	p.Glu81=	1	White	AoU	n.p.	n.p.
109	rs1455947059	c.244T>C	p.Asn82Asp	1	African	AoU	Neutral	75
110	n.a.	c.244T>A	p.Asn82Tyr	1	White	AoU	Deleterious	55
111	rs1211418416	c.246A>G	p.Asn82=	2	African	AoU	n.p.	n.p.
				1	Latin American	AoU		
112	rs748115236	c.248T>C	p.Glu83Gly	8	African	AoU	Neutral	63
113	VCV002830558.1	c.250G>A	p.Leu84=	n.a.	n.a.	ClinVar	n.p.	n.p.
114	rs1853483590<br>VCV001040668.7	c.251A>T	p.Leu84Gln	n.a.	n.a.	ClinVar	Deleterious	87
115	VCV002862695.1	c.252T>C	p.Leu84=	n.a.	n.a.	ClinVar	n.p.	n.p.
116	rs1205512895	c.254G>A	p.Thr85Met	1	Asian	AoU	Deleterious	55
				2	White	AoU		
117	rs534375493	c.255C>T	p.Thr85=	1	Asian	AoU	n.p.	n.p.
				2	White	AoU		
				1	Latin American	AoU		
118	rs534375493	c.255C>A	p.Thr85=	1	Asian	GA100K	n.p.	n.p.
				1	Asian	1000GP		
119	n.a.	c.259A>G	p.Tyr87His	2	African	AoU	Neutral	75
120	rs571834768	c.259A>C	p.Tyr87Asp	1	African	1000GP	Neutral	63
121	VCV002856963.1	c.261G>A	p.Tyr87=	n.a.	n.a.	ClinVar	n.p.	n.p.

122	n.a.	c.262A>G	p.Cys88Arg	1	Latin American	AoU	Deleterious	87
123	n.a.	c.265A>G	p.Cys89Arg	1	White	AoU	Deleterious	87
124	rs397514767	c.266C>T	p.Cys89Tyr	1	White	1000GP	Deleterious	87
				14	African	[11,12]		
	VCV000064690.2			n.a.	n.a.	ClinVar		
125	rs1384972145	c.267G>A	p.Cys89=	6	African	AoU	n.p.	n.p.
				1	Unknown	AoU		
126	n.a.	c.269T>C	p.Lys90Arg	1	Latin American	AoU	Neutral	83
127	rs1853482212	c.271T>C	p.Lys91Glu	1	Unknown	AoU	Neutral	65
128	rs758095639	c.274C>T	p.Asp92Asn	1	African	AoU	Neutral	72
				4	White	AoU		
				1	Asian	AoU		
				1	Unknown	AoU		
129	rs758095639	c.274C>A	p.Asp92Tyr	1	Latin American	AoU	Deleterious	78
130	rs750005211	c.278A>G	p.Leu93Pro	3	Latin American	AoU	Deleterious	87
131	rs1323755929	c.285delG	p.Phe96fs	5	White	AoU	n.p.	n.p.
				1	Unknown	AoU		
132	VCV002585022.1	c.286_295del	p.Phe96fs	n.a.	n.a.	ClinVar	n.p.	n.p.
133	rs200170584	c.286A>G	p.Phe96Leu	3	White	AoU	Neutral	74
	VCV001354058.7			n.a.	n.a.	ClinVar		
134	rs200170584	c.288A>G	p.Phe96=	2	African	AoU	n.p.	n.p.
				8	White	AoU		
				1	White	1000GP		
135	VCV001354058.5	c.288A>C	p.Phe96Leu	n.a.	n.a.	ClinVar	Neutral	74
136	rs1430703499	c.290T>A	p.Asn97Ile	1	White	AoU	Deleterious	65
137	rs531865023	c.291G>A	p.Asn97=	4	African	AoU		
				2	Asian	AoU		
				4	White	AoU		
				3	Latin American	AoU		
				3	Unknown	AoU		
				1	Asian	1000GP		
				1	Asian	WGP		
138	rs754311428	c.292C>T	p.Glu98Lys	2	African	AoU	Neutral	65
				1	White	AoU		
				1	Asian	AoU		
				1	Unknown	AoU		
139	VCV001928026.2	c.292C>G	p.Glu98Gln	n.a.	n.a.	ClinVar	Neutral	83
140	n.a.	c.295G>C	p.Gln99Glu	1	Asian	AoU	Neutral	74
141	rs764645328	c.298G>A	p.Leu100Phe	5	White	AoU	Neutral	65
142	rs761008545	c.299A>C	p.Leu100Arg	16	White	AoU	Deleterious	51

				1	Unknown	AoU		
143	rs971099362	c.301C>T	p.Glu101Lys	5	White	AoU	Neutral	74
144	VCV003599565.1	c.301delC	p.Glu101fs	n.a.	n.a.	ClinVar	n.p.	n.p.
145	VCV001999350.2	c.302T>G	p.Glu101Ala	n.a.	n.a.	ClinVar	Neutral	75
146	rs201067115	c.313T>G	p.Thr105Pro	4	Asian	AoU	Deleterious	61
				7	White	AoU		
147	VCV002844243.1	c.318C>G	p.Ser106=	n.a.	n.a.	ClinVar	n.p.	n.p.
148	VCV002904017.1	c.319A>G	p.Leu107=	n.a.	n.a.	ClinVar	n.p.	n.p.
149	rs749308157	c.323G>T	p.Ser108Ter	1	Asian	1000GP	n.p.	n.p.
				2	White	[13]		
150	VCV002110523.1	c.328T>G	p.Lys110Gln	n.a.	n.a.	ClinVar	Deleterious	61
151	VCV002979265.1	c.333A>G	p.Thr111=	n.a.	n.a.	ClinVar	n.p.	n.p.
152	rs764428686	c.336delGAA	p.Leu115del	47	African	AoU	n.p.	n.p.
				1	Latin American	AoU		
				1	Unknown	AoU		
153	n.a.	c.338A>G	p.Leu113Pro	1	Asian	IndiGen	Deleterious	87
154	rs563206374	c.342C>A	p.Leu114=	1	African	1000GP	n.p.	n.p.
155	VCV001400491.5	c.360C>T	p.Leu120=	n.a.	n.a.	ClinVar	n.p.	n.p.
156	VCV002159226.1	c.361C>A	p.Ala121Ser	n.a.	n.a.	ClinVar	Neutral	65
157	n.a.	c.361delC	p.Ala121fs	1	Asian	[3,4]	n.p.	n.p.
158	VCV002970441.1	c.366A>C	p.Ala122=	n.a.	n.a.	ClinVar	n.p.	n.p.
159	rs369200735	c.382G>A	p.Pro128Ser	23	White	AoU	Deleterious	61
				4	Latin American	AoU		
				4	Unknown	AoU		
160	rs549099946	c.384G>T	p.Pro128=	1	Asian	1000GP	n.p.	n.p.

† Relative to NCBI reference sequence NM_203330.2
‡ Relative to NCBI reference sequence NP_976075.1
§ "Unknown" refers to "not specified"
⁋ Normalized confidence as calculated by PredictSNP
AoU, All of Us; IndiGen, The IndiGenomes database; GA100K, The GenomeAsia 100K Project; 1000GP, The 1000 Genomes Project
n.a.─ not available; n.p.─ not applicable

References

1. Chai JN, Azad AK, Kuan K, Guo X, Wang Y. A splice site mutation associated with congenital CD59 deficiency. Hematol Rep 2022; 14: 172-178. doi: 10.3390/hematolrep14020025

2. Javadi Parvaneh V, Ghasemi L, Rahmani K, Shiari R, Mesdaghi M, Chavoshzadeh Z, et al. Recurrent angioedema, Guillain-Barré, and myelitis in a girl with systemic lupus erythematosus and CD59 deficiency syndrome. Auto Immun Highlights 2020; 11: 9. doi: 10.1186/s13317-020-00132-2

3. Yamashina M, Ueda E, Kinoshita T, Takami T, Ojima A, Ono H, et al. Inherited complete deficiency of 20-kilodalton homologous restriction factor (CD59) as a cause of paroxysmal nocturnal hemoglobinuria. N Engl J Med 1990; 323: 1184-1189. doi: 10.1056/nejm199010253231707

4. Motoyama N, Okada N, Yamashina M, Okada H. Paroxysmal nocturnal hemoglobinuria due to hereditary nucleotide deletion in the HRF20 (CD59) gene. Eur J Immunol 1992; 22: 2669-2673. doi: 10.1002/eji.1830221029

5. Haliloglu G, Maluenda J, Sayinbatur B, Aumont C, Temucin C, Tavil B, et al. Early-onset chronic axonal neuropathy, strokes, and hemolysis: inherited CD59 deficiency. Neurology 2015; 84: 1220-1224. doi: 10.1212/wnl.0000000000001391

6. Yuksel D, Oguz KK, Azapagası E, Kesici S, Cavdarli B, Konuskan B, et al. Uncontrolled inflammation of the nervous system: Inherited CD59 deficiency. Neurol Clin Pract 2018; 8: e18-e20. doi: 10.1212/cpj.0000000000000511

7. Klemann C, Kirschner J, Ammann S, Urbach H, Moske-Eick O, Zieger B, et al. CD59 deficiency presenting as polyneuropathy and Moyamoya syndrome with endothelial

abnormalities of small brain vessels. Eur J Paediatr Neurol 2018; 22: 870-877. doi: 10.1016/j.ejpn.2018.04.003

8. Höchsmann B, Dohna-Schwake C, Kyrieleis HA, Pannicke U, Schrezenmeier H. Targeted therapy with eculizumab for inherited CD59 deficiency. N Engl J Med 2014; 370: 90-92. doi: 10.1056/NEJMc1308104

9. Ardicli D, Taskiran EZ, Kosukcu C, Temucin C, Oguz KK, Haliloglu G, et al. Neonatal-onset recurrent Guillain-Barré Syndrome-like disease: clues for inherited CD59 deficiency. Neuropediatrics 2017; 48: 477-481. doi: 10.1055/s-0037-1604483

10. Li XF, Lin FQ, Li JP. Identification of c.238 A>G (p.Arg80Gly) of CD59 blood group gene. Transfusion 2018; 58: 3033-3034. doi: 10.1111/trf.14960

11. Nevo Y, Ben-Zeev B, Tabib A, Straussberg R, Anikster Y, Shorer Z, et al. CD59 deficiency is associated with chronic hemolysis and childhood relapsing immune-mediated polyneuropathy. Blood 2013; 121: 129-135. doi: 10.1182/blood-2012-07-441857

12. Ben-Zeev B, Tabib A, Nissenkorn A, Garti BZ, Gomori JM, Nass D, et al. Devastating recurrent brain ischemic infarctions and retinal disease in pediatric patients with CD59 deficiency. Eur J Paediatr Neurol 2015; 19: 688-693. doi: 10.1016/j.ejpn.2015.07.001

13. Solmaz I, Aytekin ES, Çağdaş D, Tan C, Tezcan I, Gocmen R, et al. Recurrent demyelinating episodes as sole manifestation of inherited CD59 deficiency. Neuropediatrics 2020; 51: 206-210. doi: 10.1055/s-0039-3399583

Table S2. *CD59* variant allele observations in different populations

		Population					
S. No.	*CD59* variant	African	White	Asian	Latin American	Unknown	Total
1	c.1T>C	1	3	0	3	0	7
2	c.2A>G			n.a.			n.a.
3	c.5C>A	0	0	0	0	1	1
4	c.11T>A	5	0	0	0	0	5
5	c.13C>T	0	1	0	0	0	1
6	c.16C>A	0	1	0	0	0	1
7	c.18C>T	85	929	2	149	78	1243
8	c.18C>A	0	3	0	0	0	3
9	c.22C>A	1	5	0	0	0	6
10	c.23A>G	0	1	0	0	0	1
11	c.30G>A	24	129	0	17	9	179
12	c.31C>T	0	7	0	0	0	7
13	c.33C>G			n.a.			n.a.
14	c.35A>C	0	1	0	0	0	1
15	c.37G>A	0	0	0	1	0	1
16	c.42G>A	0	1	0	2	0	3
17	c.43C>T	1	6	0	1	1	9
18	c.43C>A	0	0	1	0	0	1
19	c.46G>C	0	0	0	1	0	1
20	c.46G>A	1	5	0	0	1	7
21	c.47A>G	0	0	0	1	0	1
22	c.49C>T	0	0	1	0	0	1
23	c.49C>A	0	1	0	0	0	1
24	c.50G>A	0	1	0	0	0	1
25	c.51A>G	0	3	0	6	0	9
26	c.52C>T	0	0	0	34	1	35
27	c.53A>C	0	0	0	2	0	2
28	c.54G>A	503	30	1	91	51	676
29	c.55A>G	0	0	0	0	1	1
30	c.59C>T	0	0	1	0	0	1
31	c.60G>C	0	0	0	0	1	1
32	c.65G>A	0	6	0	0	0	6
33	c.67C>T	0	0	2	0	0	2
34	c.67+1C>A	0	2	0	0	0	2
35	c.68-2T>C	2	0	0	0	0	2
36	c.70G>C	0	4	0	0	0	4
37	c.70G>A	0	1	0	2	0	3
38	c.71T>C	9	26	1	2	8	46
39	c.72A>G	6	91	0	103	11	211
40	c.76G>A	2	0	0	0	0	2
41	c.83C>T	0	0	0	?	1	1
42	c.84G>C	1	0	0	0	0	1
43	c.85A>C	0	2	0	0	0	2
44	c.88T>A	0	0	0	0	1	1
45	c.89T>C	1	0	1	0	0	2
46	c.89T>G	0	0	1	0	0	1

47	c.92C>T	0	1	0	0	0	1
48	c.98T>G	2	27	0	3	0	32
49	c.99G>C	0	1	0	4	0	5
50	c.99G>A	1	4	1	2	0	8
51	c.109C>T	2	0	0	0	0	2
52	c.118T>A	0	0	1	0	0	1
53	c.119G>A	9	0	0	0	0	9
54	c.120T>A			n.a.			n.a.
55	c.123G>A	6	1	0	0	0	7
56	c.123delG	0	0	1	0	0	1
57	c.124C>T	1	4	1	6	3	15
58	c.126G>C	2004	12	3	163	129	2311
59	c.127T>G	0	1	0	0	0	1
60	c.127T>A	1	0	0	0	0	1
61	c.128T>C	0	0	0	1	0	1
62	c.129A>G	0	5	0	0	1	6
63	c.132A>G	0	1	0	0	0	1
64	c.135T>A	0	0	0	3	0	3
65	c.143_144delAA			n.a.			n.a.
66	c.145C>G	3	0	0	0	0	3
67	c.146T>A	0	4	0	0	1	9
68	c.146delT	0	6	0	0	0	6
69	c.148C>T	4	0	0	0	0	4
70	c.149G>A	0	11	1	11	2	25
71	c.150C>T	101	340	7	137	36	621
72	c.153A>G	0	1	0	0	0	1
73	c.155A>G	0	1	0	0	0	1
74	c.156G>C	0	5	0	0	0	5
75	c.156G>A	0	0	8	0	0	8
76	c.162G>C	0	1	0	0	0	1
77	c.165T>C			n.a.			n.a.
78	c.168A>G	1	0	0	0	0	1
79	c.169+1C>G	0	0	1	2	0	3
80	c.171C>T	63	19	0	0	4	86
81	c.171C>A	0	0	0	1	0	1
82	c.179A>G	0	7	0	0	0	7
83	c.180C>T	0	0	0	1	0	1
84	c.183_184dup			n.a.			n.a.
85	c.189C>T	0	0	0	1	0	1
86	c.190A>C			n.a.			n.a.
87	c.190delA	3	0	0	0	0	3
88	c.192A>G	0	0	1	0	0	1
89	c.195C>G	0	0	0	0	1	1
90	c.198C>T	4	7	0	0	0	11
91	c.202C>G	0	1	0	0	0	1
92	c.204C>T	1	0	0	0	0	1
93	c.214A>C	0	1	0	0	0	1
94	c.216G>C	0	1	0	0	0	1
95	c.219G>A	1	8	0	0	1	10
96	c.220C>T	2	2	0	2	0	6
97	c.222G>A	850	7	0	2	57	916
98	c.223C>T	1	2	0	1	1	5
99	c.230G>T	1	0	0	0	0	1
100	c.230G>A	0	2	0	0	0	2

101	c.231G>A			n.a.			n.a.
102	c.232G>A	0	2	0	1	0	3
103	c.233C>T	0	2	0	1	0	3
104	c.233C>G			n.a.			n.a.
105	c.235A>G			n.a.			n.a.
106	c.238T>C			n.a.			n.a.
107	c.241C>T	0	1	0	0	0	1
108	c.243T>C	0	1	0	0	0	1
109	c.244T>C	1	0	0	0	0	1
110	c.244T>A	0	1	0	0	0	1
111	c.246A>G	2	0	0	1	0	3
112	c.248T>C	8	0	0	0	0	8
113	c.250G>A			n.a.			n.a.
114	c.251A>T			n.a.			n.a.
115	c.252T>C			n.a.			n.a.
116	c.254G>A	0	2	1	0	0	3
117	c.255C>T	0	2	1	1	0	4
118	c.255C>A	0	0	2	0	0	2
119	c.259A>G	2	0	0	0	0	2
120	c.259A>C	1	0	0	0	0	1
121	c.261G>A			n.a.			n.a.
122	c.262A>G	0	0	0	1	0	1
123	c.265A>G	0	1	0	0	0	1
124	c.266C>T	14	1	0	0	0	15
125	c.267G>A	6	0	0	0	1	7
126	c.269T>C	0	0	0	1	0	1
127	c.271T>C	0	0	0	0	1	1
128	c.274C>T	1	4	1	0	1	7
129	c.274C>A	0	0	0	1	0	1
130	c.278A>G	0	0	0	3	0	3
131	c.285delG	0	5	0	0	1	6
132	c.286_295del			n.a.			n.a.
133	c.286A>G	0	3	0	0	0	3
134	c.288A>G	2	9	0	0	0	11
135	c.288A>C			n.a.			n.a.
136	c.290T>A	0	1	0	0	0	1
137	c.291G>A	4	4	4	3	3	18
138	c.292C>T	2	1	1	0	1	5
139	c.292C>G			n.a.			n.a.
140	c.295G>C	0	0	1	0	0	1
141	c.298G>A	0	5	0	0	0	5
142	c.299A>C	0	16	0	0	1	17
143	c.301C>T	0	5	0	0	0	5
144	c.301delC			n.a.			n.a.
145	c.302T>G			n.a.			n.a.
146	c.313T>G	0	7	4	0	0	11
147	c.318C>G			n.a.			n.a.
148	c.319A>G			n.a.			n.a.
149	c.323G>T	0	2	1	0	0	3
150	c.328T>G			n.a.			n.a.
151	c.333A>G			n.a.			n.a.
152	c.335_341delGAA	47	0	0	1	1	49
153	c.338A>G	0	0	1	0	0	1
154	c.342C>A	1	0	0	0	0	1

155	c.360C>T			n.a.			n.a.
156	c.361C>A			n.a.			n.a.
157	c.361delC	0	0	1	0	0	1
158	c.366A>C			n.a.			n.a.
159	c.382G>A	0	23	0	4	4	31
160	c.384G>T	0	0	1	0	0	1

n.a.─ not available in the ClinVar database

Table S3. Distribution of 91 amino acid changes in the CD59 protein

			Observations							
			Variants*				Individuals with variants*			
				Normalized				Normalized		
CD59 preproprotein segment	Amino acids	%	Observed (n)	(n)	Relative %	*P*	Observed (n)	(n)	Relative %	*P*
Signal peptide	25	19.5%	26	18	144%		146	83	176%	
						0.4131				0.0103
Mature protein	77	60.2%	60	55	109%		236	256	93%	
						0.0041				0.0353
GPI signal sequence	26	20.3%	5	18	28%		43	86	50%	
Total	128	100%	91	91			425	425		

* Comparing signal peptide versus GPI signal sequence, the number of variants (*P*=0.0021) and individuals with variants (*P*=0.0002) differed statistically significant, Fisher's exact test, two-sided.

Table S4. *CD59* variants reported in patients[1-13]

Location	Amino acid substitution*	Zygosity	Individuals observed†	PredictSNP classification	Reference	Clinical condition
Non-synonymous						
Exon 4	p.Met1Thr	n.r.	1	Deleterious	ClinVar[1]	Primary CD59 deficiency
Exon 5	p.Cys28Tyr	n.r.	1	Deleterious	ClinVar	Primary CD59 deficiency
			1		AoU	n.p.
	p.Tyr29Asp	Homozygous	1	Deleterious	2	CD59 deficiency syndrome
			1		ClinVar	Primary CD59 deficiency
	p.Asp49Val	Homozygous	4	Deleterious	3-7	CD59 deficiency syndrome
			1		AoU	n.p.
Exon 6	p.Cys64Gly	n.r.	1	Deleterious	ClinVar	Primary CD59 deficiency
	p.Arg78Pro	n.r.	1	Deleterious	ClinVar	n.p.
	p.Arg78His	n.r.	1	Neutral	ClinVar	n.p.
	p.Leu84Gln	n.r.	1	Deleterious	ClinVar	n.p.
	p.Cys89Tyr	Homozygous	7	Deleterious	8,9	Primary CD59 deficiency
			1		ClinVar	n.p.
			1		1000GP	n.p.
	p.Phe96Leu	n.r.	1	Neutral	ClinVar	n.p.
			3		AoU	n.p.
	p.Glu98Gln	n.r.	1	Neutral	ClinVar	n.p.
	p.Glu101Ala	n.r.	1	Neutral	ClinVar	n.p.
	p.Lys110Gln	n.r.	1	Deleterious	ClinVar	n.p.
	p.Ala121Ser	n.r.	1	Neutral	ClinVar	n.p.
Splice-site						
Intron 4	c.67+1C>A	Homozygous	1	n.a.	10	CD59 deficiency syndrome

Frameshift						
Exon 5	p.Ala41fs	Heterozygous	1	n.a.	11,12	Paroxysmal nocturnal hemoglobinuria
	p.Asp49fs	Homozygous	3	n.a.	5-7	CD59 deficiency syndrome
Exon 6	p.Asn62fs	n.r.	1	n.a.	ClinVar	n.p.
	p.Ala121fs	Heterozygous	1	n.a.	11,12	Paroxysmal nocturnal hemoglobinuria
	p.Phe96fs	n.r.	1	n.a.	ClinVar	Primary CD59 deficiency
	p.Glu101fs	n.r.	1	n.a.	ClinVar	Primary CD59 deficiency
Nonsense						
Exon 5	p.Phe48Ter	n.r.	1	n.a.	ClinVar	Primary CD59 deficiency
Exon 6	p.Ser108Ter	Homozygous	1	n.a.	13	CD59 deficiency syndrome
			1		1000GP	n.p.
Synonymous						
Exon 4	p.Gly11=	n.r.	1	n.a.	ClinVar	n.p.
Exon 5	p.Thr40=	n.r.	1	n.a.	ClinVar	n.p.
	p.Lys55=	n.r.	1	n.a.	ClinVar	n.p.
Exon 6	p.Thr77=	n.r.	1	n.a.	ClinVar	n.p.
	p.Leu79=	n.r.	1	n.a.	ClinVar	n.p.
	p.Leu84=	n.r.	1	n.a.	ClinVar	n.p.
	p.Tyr87=	n.r.	1	n.a.	ClinVar	n.p.
	p.Ser106=	n.r.	1	n.a.	ClinVar	n.p.
	p.Leu107=	n.r.	1	n.a.	ClinVar	n.p.
	p.Thr111=	n.r.	1	n.a.	ClinVar	n.p.
	p.Leu120=	n.r.	1	n.a.	ClinVar	n.p.
	p.Ala122=	n.r.	1	n.a.	ClinVar	n.p.
Total			55			

* see Table S1

† not all ClinVar submissions report the number of individuals observed and are set to n=1 in the current table.

AoU, All of Us database; 1000GP, The 1000 Genomes Project

n.a. — not applicable; n.r. — not reported; n.p. — not provided

References

1. Landrum MJ, Lee JM, Benson M, Brown GR, Chao C, Chitipiralla S, et al. ClinVar: improving access to variant interpretations and supporting evidence. Nucleic Acids Res 2018;46:D1062-d7.
2. Javadi Parvaneh V, Ghasemi L, Rahmani K, Shiari R, Mesdaghi M, Chavoshzadeh Z, et al. Recurrent angioedema, Guillain-Barré, and myelitis in a girl with systemic lupus erythematosus and CD59 deficiency syndrome. Auto Immun Highlights 2020;11:9.
3. Haliloglu G, Maluenda J, Sayinbatur B, Aumont C, Temucin C, Tavil B, et al. Early-onset chronic axonal neuropathy, strokes, and hemolysis: inherited CD59 deficiency. Neurology 2015;84:1220-4.
4. Yuksel D, Oguz KK, Azapagası E, Kesici S, Cavdarli B, Konuskan B, et al. Uncontrolled inflammation of the nervous system: Inherited CD59 deficiency. Neurol Clin Pract 2018;8:e18-e20.
5. Klemann C, Kirschner J, Ammann S, Urbach H, Moske-Eick O, Zieger B, et al. CD59 deficiency presenting as polyneuropathy and Moyamoya syndrome with endothelial abnormalities of small brain vessels. Eur J Paediatr Neurol 2018;22:870-7.
6. Höchsmann B, Dohna-Schwake C, Kyrieleis HA, Pannicke U, Schrezenmeier H. Targeted therapy with eculizumab for inherited CD59 deficiency. N Engl J Med 2014;370:90-2.

7. Ardicli D, Taskiran EZ, Kosukcu C, Temucin C, Oguz KK, Haliloglu G, et al. Neonatal-onset recurrent Guillain-Barré Syndrome-like disease: clues for inherited CD59 deficiency. Neuropediatrics 2017;48:477-81.

8. Nevo Y, Ben-Zeev B, Tabib A, Straussberg R, Anikster Y, Shorer Z, et al. CD59 deficiency is associated with chronic hemolysis and childhood relapsing immune-mediated polyneuropathy. Blood 2013;121:129-35.

9. Ben-Zeev B, Tabib A, Nissenkorn A, Garti BZ, Gomori JM, Nass D, et al. Devastating recurrent brain ischemic infarctions and retinal disease in pediatric patients with CD59 deficiency. Eur J Paediatr Neurol 2015;19:688-93.

10. Chai JN, Azad AK, Kuan K, Guo X, Wang Y. A splice site mutation associated with congenital CD59 deficiency. Hematol Rep 2022;14:172-8.

11. Yamashina M, Ueda E, Kinoshita T, Takami T, Ojima A, Ono H, et al. Inherited complete deficiency of 20-kilodalton homologous restriction factor (CD59) as a cause of paroxysmal nocturnal hemoglobinuria. N Engl J Med 1990;323:1184-9.

12. Motoyama N, Okada N, Yamashina M, Okada H. Paroxysmal nocturnal hemoglobinuria due to hereditary nucleotide deletion in the HRF20 (CD59) gene. Eur J Immunol 1992;22:2669-73.

13. Solmaz I, Aytekin ES, Çağdaş D, Tan C, Tezcan I, Gocmen R, et al. Recurrent demyelinating episodes as sole manifestation of inherited CD59 deficiency. Neuropediatrics 2020;51:206-10.

Table S5. PredictSNP classified deleterious non-synonymous *CD59* variants

S. No.	Nucleotide	Amino acid	Alleles observed	Population	PredictSNP expected accuracy (%)	Function	Potential or known variant impact
1	c.1T>C	p.Met1Val	1<br>3<br>3	African<br>White<br>Latin American	51	Start codon	Loss of protein synthesis
2	c.2A>G	p.Met1Thr	n.a.	n.a.	51	Start codon	Loss of protein synthesis
3	c.5C>A	p.Gly2Val	1	Unknown	76	Located in signal peptide	Affect protein targeting, translocation, processing, and stability
4	c.11T>A	p.Gln4Leu	5	African	51	Located in signal peptide	Affect protein targeting, translocation, processing, and stability
5	c.13C>T	p.Gly5Arg	1	White	87	Located in signal peptide	Affect protein targeting, translocation, processing, and stability
6	c.16C>A	p.Gly6Trp	1	White	76	Located in signal peptide	Affect protein targeting, translocation, processing, and stability
7	c.22C>A	p.Val8Phe	1<br>5	African<br>White	65	Located in signal peptide	Affect protein targeting, translocation, processing, and stability
8	c.31C>T	p.Gly11Arg	7	White	72	Located in signal peptide	Affect protein targeting, translocation, processing, and stability
9	c.35A>C	p.Leu12Arg	1	White	87	Located in signal peptide	Affect protein targeting, translocation, processing, and stability
10	c.47A>G	p.Leu16Pro	1	Latin American	97	Located in signal peptide	Affect protein targeting, translocation, processing, and stability
11	c.49C>T	p.Ala17Thr	1	Asian	60	Located in signal peptide	Affect protein targeting, translocation, processing, and stability
12	c.50G>A	p.Ala17Val	1	White	51	Located in signal peptide	Affect protein targeting, translocation, processing, and stability
13	c.53A>C	p.Val18Gly	2	Latin American	87	Located in signal peptide	Affect protein targeting, translocation, processing, and stability
14	c.59C>T	p.Cys20Tyr	1	Asian	61	Located in signal peptide	Affect protein targeting, translocation, processing, and stability
15	c.60G>C	p.Cys20Trp	1	Unknown	87	Located in signal peptide	Affect protein targeting, translocation, processing, and stability
16	c.83C>T	p.Cys28Tyr	1	Unknown	87	Forms disulfide linkage	Destabilizes protein structure
17	c.84G>C	p.Cys28Trp	1	African	87	Forms disulfide linkage	Destabilizes protein structure

18	c.85A>C	p.Tyr29Asp	2	White	87	Unknown	Unknown
19	c.92C>T	p.Cys31Tyr	1	White	87	Forms disulfide linkage	Loss of disulfide linkage
20	c.127T>G	p.Asn43His	1	White	72	N-glycosylation site	None
21	c.127T>A	p.Asn43Tyr	1	African	51	N-glycosylation site	None
22	c.145C>G	p.Asp49His	3	African	87	MAC inhibitory function	Affect complement-inhibitory activity
23	c.146T>A	p.Asp49Val	8	White	87	MAC inhibitory function	Affect complement-inhibitory activity
24	c.155A>G	p.Leu52Pro	1	White	87	MAC inhibitory function	Affect complement-inhibitory activity
25	c.190A>C	p.Cys64Gly	n.a.	n.a.	87	MAC inhibitory function	Affect complement-inhibitory activity
26	c.195C>G	p.Trp65Cys	1	Unknown	87	MAC inhibitory function	Affect complement-inhibitory activity
27	c.232G>A	p.Arg78Cys	1	White	76	MAC inhibitory function	Affect complement-inhibitory activity
			1	Latin American			
			1	White			
28	c.233C>G	p.Arg78Pro	1	ClinVar	61	MAC inhibitory function	Affect complement-inhibitory activity
29	c.241C>T	p.Glu81Lys	1	White	87	MAC inhibitory function	Affect complement-inhibitory activity
30	c.244T>A	p.Asn82Tyr	1	White	55	MAC inhibitory function	Affect complement-inhibitory activity
31	c.251A>T	p.Leu84Gln	1	ClinVar	87	Unknown	Unknown
32	c.254G>A	p.Thr85Met	1	Asian	55	O-glycosylation site	Unknown
			2	White			
33	c.262A>G	p.Cys88Arg	1	Latin American	87	Forms disulfide linkage	Destabilizes protein structure
34	c.265A>G	p.Cys89Arg	1	White	87	Forms disulfide linkage	Destabilizes protein structure
35	c.266C>T	p.Cys89Tyr	1	White	87	Forms disulfide linkage	Destabilizes protein structure
			14	African American			
36	c.274C>A	p.Asp92Tyr	1	Latin American	78	Unknown	Unknown
37	c.278A>G	p.Leu93Pro	3	Latin American	87	Unknown	Unknown
38	c.290T>A	p.Asn97Ile	1	White	65	Unknown	Unknown
39	c.299A>C	p.Leu100Arg	16	White	51	Unknown	Unknown
			1	Unknown			
40	c.313T>G	p.Thr105Pro	4	Asian	61	GPI signal sequence	Altered GPI-anchor translocation
			7	White			
41	c.328T>G	p.Lys110Gln	1	ClinVar	61	GPI signal sequence	Altered GPI-anchor translocation
42	c.338A>G	p.Leu113Pro	1	Asian	87	GPI signal sequence	Altered GPI-anchor translocation
43	c.382G>A	p.Pro128Ser	23	White	61	GPI signal sequence	Altered GPI-anchor translocation
			4	Latin American			
			4	Unknown			

n.a.– not available

Table S6. Estimated prevalence of *CD59* deleterious variants in White population

		Estimated prevalence of an individual in the population		
Variant	Alleles observed	Heterozygous for the deleterious allele	Homozygous for the deleterious allele	Homozygous for the deleterious allele after inbreeding correction
c.1T>C	3	1 in 85,770	1 in 3,678,246,450	1 in 36,602,366
c.13C>T	1	1 in 257,310	1 in 33,104,218,050	1 in 110,539,570
c.16C>A	1	1 in 257,310	1 in 33,104,218,050	1 in 110,539,570
c.22C>A	5	1 in 51,462	1 in 1,324,168,722	1 in 21,816,854
c.31C>T	7	1 in 36,759	1 in 675,596,287	1 in 15,481,557
c.35A>C	1	1 in 257,310	1 in 33,104,218,050	1 in 110,539,570
c.50G>A	1	1 in 257,310	1 in 33,104,218,050	1 in 110,539,570
c.92C>T	1	1 in 257,310	1 in 33,104,218,050	1 in 110,539,570
c.127T>G	1	1 in 257,310	1 in 33,104,218,050	1 in 110,539,570
c.155A>G	1	1 in 257,310	1 in 33,104,218,050	1 in 110,539,570
c.232G>A	2	1 in 128,655	1 in 8,276,054,513	1 in 55,086,059
c.241C>T	1	1 in 257,310	1 in 33,104,218,050	1 in 110,539,570
c.244T>A	1	1 in 257,310	1 in 33,104,218,050	1 in 110,539,570
c.254G>A	2	1 in 128,655	1 in 8,276,054,513	1 in 55,086,059
c.265A>G	1	1 in 257,310	1 in 33,104,218,050	1 in 110,539,570
c.290T>A	1	1 in 257,310	1 in 33,104,218,050	1 in 110,539,570
c.299A>C	16	1 in 16,082	1 in 129,313,352	1 in 6,579,555
c.313T>G	7	1 in 36,759	1 in 675,596,287	1 in 15,481,557
c.382G>A	23	1 in 11,187	1 in 62,578,862	1 in 4,477,526
